# Chlorophyllin exerts synergistic anti-tumor effect with gemcitabine in pancreatic cancer by inducing cuproptosis

**DOI:** 10.1186/s10020-025-01180-y

**Published:** 2025-04-04

**Authors:** Jiaqiang Ren, Tong Su, Jiachun Ding, Fan Chen, Jiantao Mo, Jie Li, Zheng Wang, Liang Han, Zheng Wu, Shuai Wu

**Affiliations:** 1 Department of Hepatobiliary Surgery, The First Affiliated Hospital of Xi’an Jiaotong University, Xi’an, Shaanxi 710061 China; 2 School of Public Health, Xi’an Jiaotong University Health Science Center, Xi’an, Shaanxi 710061 China

**Keywords:** Pancreatic cancer, Chlorophyllin, Gemcitabine, Synergistic effect, Cuproptosis

## Abstract

Pancreatic cancer (PC) has high lethality due to multiple reasons, and its limited response to conventional chemotherapy like gemcitabine (GEM) is a non-negligible one. Therefore, our study introduces Chlorophyllin (CHL) as an effective therapeutic candidate to enhance the therapeutic efficacy of GEM. Our results demonstrate that the combination of CHL and GEM exhibits a significant synergistic anti-tumor effect by targeting multiple oncogenic processes in PC, including inhibiting cell proliferation, invasion, and migration, as well as inducing cell apoptosis. Further investigations of mechanism have revealed that CHL induces cuproptosis in PC cells through a multifaceted process, involving depleting cellular intracellular glutathione (GSH), increasing reactive oxygen species (ROS) levels, and subsequently upregulating the HSP70 protein in response to heightened oxidative stress. Additionally, CHL releases free Cu^2+^, binds to the Ferredoxin 1 (FDX1) protein, and ultimately leads to the oligomerization of Dihydrolipoamide S-Acetyltransferase (DLAT) proteins to amplify the copper toxicity within PC cells. Moreover, in vivo experiments have demonstrated that the combination of CHL and GEM effectively inhibits the growth of subcutaneously transplanted tumors while maintaining a favorable biosafety profile. In conclusion, our study identifies CHL as a potent enhancer of GEM’s anti-tumor effects in PC through the induction of cuproptosis, thus providing a novel therapeutic avenue for patients with PC.

**Supplementary Information:**

The online version contains supplementary material available at 10.1186/s10020-025-01180-y.

## Introduction

Pancreatic cancer (PC) is one of the most malignant types of cancer worldwide, with a 5-year survival rate of only about 13% (Siegel et al. 2024). Its lethality is underscored by the fact that it is currently the third-leading cause of cancer death in the United States, and is projected to become the second by 2040 (Rahib et al. 2021). One of the main reasons behind the high mortality rate of PC is the difficulty in detecting the disease in its early stages, leading to the progression of distant metastases (Halbrook et al. 2023). Consequently, only 15–20% of patients are eligible for surgery at the time of diagnosis, making chemotherapy the primary treatment option for patients at all stages of PC (Kleeff et al. 2016; Mizrahi et al. 2020). The initial sensitivity of PC to chemotherapeutic agents provides patients with an opportunity for surgical treatment.

Gemcitabine (GEM) has served as a cornerstone and first-line chemotherapy drug for PC and being widely utilized in clinical practice (Kipps et al. 2017). Despite its established efficacy, PC patients undergoing GEM treatment frequently face challenges associated with primary and secondary chemotherapy resistance, which significantly curtail the effectiveness of therapy (Schultheis et al. 2017; Ren et al. 2024). Combination regimens incorporating various chemotherapeutic agents can partially enhance treatment efficacy, however, they often result in significantly increased toxicities that may be intolerable for patients with PC (Hoff et al. 2013). Resistance to GEM in PC is partially attributed to the disturbance of intracellular redox balance. Therefore, the introduction of modalities that induce cell death by elevating cellular reactive oxygen species (ROS) levels, such as ferroptosis (Yang et al. 2021), has demonstrated a more potent therapeutic effect on drug-resistant cells.

Cuproptosis was first introduced by Tsvetkov (Tsvetkov et al. 2022) and colleagues in 2022, which is a novel form of regulated, copper-induced cell death. Cuproptosis is primarily triggered by the accumulation of monovalent Cu (Cu^+^) in mitochondria, which results in the alterations in cellular redox state, aggregation of lipoylated Dihydrolipoamide S-Acetyltransferase (DLAT), causing proteotoxic stress and ultimately leading to cell death (Cobine et al. 2022). Studies have demonstrated that cancer patients exhibit significantly higher levels of copper in both tumor tissue and serum compared to healthy individuals, with elevated copper levels being toxic to cells (Oliveri 2022; Kamiya 2022). The emergence of cuproptosis as a well-defined concept has garnered significant attention of cancer researchers, as the studies have demonstrated that that copper-based therapies play a crucial role in inhibiting tumor growth (Yu et al. 2023; Xu et al. 2023). Consequently, there is growing interest in exploring whether novel compounds that induces cuproptosis could provide new therapeutic avenues for the treatment of PC, particularly in cases that are resistant to conventional chemotherapy such as GEM.

Chlorophyllin (CHL), a sodium-copper salt of derived from chlorophyll, is primarily employed as a food additive and clinically as a wound healing enhancer or anti-inflammatory agent (Hayes and Ferruzzi 2020). Previous studies have documented the antigenotoxic, antioxidant, and anticancer properties of CHL, both in vitro and in vivo (Abdel-Latif et al. 2017; Vankova et al. 2018). As the mechanisms, CHL has been observed to increase the level of cellular ROS that facilitate cancer cell death (Sun et al. 2021). Simultaneously, CHL has been shown to directly modulate the redox environment of cancer cells, thereby disrupting the functionality of various tumor cells including breast cancer (Ozcan et al. 2021)and PC (Vankova et al. 2018). Furthermore, CHL releases free copper ions, which may induce copper-mediated toxicity within cells (Niu et al. 2021). Nevertheless, CHL’s potential to increase antitumoral effects of chemotherapeutic drugs in PC warrants further exploration.

In this study, our aim is to investigate the effects of combining CHL with GEM in treating PC, alongside elucidating its underlying mechanism of action. Our findings demonstrated a significant synergistic anti-tumor effect in PC with the combined treatment of CHL and GEM. Evidence was demonstrated through the inhibition of cell proliferation, colony formation, invasion, and migration, as well as the promotion of cell apoptosis. Subsequent analysis revealed that the therapeutic effect of CHL is mediated by the induction of cuproptosis in PC cells by the elevation of intracellular divalent copper ion (Cu^2+^) levels, modulation of the cellular redox state, and interaction with Ferredoxin 1 (FDX1). Moreover, our in vivo experiments confirmed the synergistic efficacy of the CHL and GEM combination, establishing the safety profile of this treatment. The observed synergistic anti-tumor effect of CHL, coupled with the favorable biosafety in our study indicates its promising clinical potential for the comprehensive treatment of PC patients, thus offering a novel therapeutic strategy.

## Materials and methods

### Cell culture

The human PC cell lines BxPC-3 and MIA PaCa-2, along with the human pancreatic ductal epithelial cell line hTERT-HPNE were purchased from the Chinese Academy of Sciences Cell Bank of Type Culture Collection (CBTCCCAS, Shanghai, China). MIA PaCa-2 and hTERT-HPNE cells were cultured in Dulbecco’s modified Eagle’s medium (DMEM; Gibco; Thermo Fisher Scientific, USA), while BxPC-3 cells were cultured in RPMI-1640 supplemented with 10% fetal bovine serum (FBS) at 37 °C with 5% CO_2_.

### Reagents

Chlorophyllin sodium copper salt (CHL), Gemcitabine (GEM), Ammonium tetrathiomolybdate(VI) (TTM) and Elesclomol (ELE) were purchased from MedChemExpress (MCE, USA). The antibodies used in this research included DLAT (13426-1-AP), HSP70 (10995-1-AP), FDX1 (12592-1-AP), β-actin (66009-1-Ig), and rabbit IgG (30000-0-AP), all of which were purchased from Proteintech (Rosemont, IL, USA). Additionally, CCK-8 was purchased from GLPBIO (Montclair, CA, USA).

### Cell viability assay

PC cells were inoculated in 96-well plates at about 2000 per well, followed by the addition of the respective drug interventions(CHL:30 µΜ)after 12 h. Subsequently, at 24 h/48 h/96 h intervals, 10µL of CCK-8 solution (10%) was added to each well. Cell viability was determined by measuring the optical density (OD) at 450 nm. The half-maximal inhibitory concentration (IC50) of the drug was then calculated using GraphPad Prism 9.1 Software (San Diego, CA, USA).

### Colony formation assay

PC cells were resuspended into single cells and seeded into 6-well plates with 1,000 cells per well, and appropriate drug interventions were administered after 12 h of culture. The cells were maintained under standard culture conditions for approximately 14 days, with a change of fresh medium every 2 days. Subsequently, the cells were fixed and stained with crystal violet, and the colonies were observed and counted under a microscope.

### Drug combination analysis

Cell interventions were conducted using various combinations of GEM and CHL at different concentrations, guided by the IC50 of 48 h specific to each cell line. Evaluation of cell activity was based on the corresponding OD (optical density) values. To determine the synergistic effect of GEM and CHL, two distinct methods were employed. Firstly, the Chou-Talalay approach (Chou and Talalay 1984) by CompuSyn program was used, with the combination index (CI) measuring the degree of synergy: synergism (CI < 1.0), additivity (1.0 < CI < 1.5) and antagonism (CI > 1.5). Secondly, the online tool SynergyFinder (https://synergyfinder.fimm.fi) was utilized, where drug synergy scoring was calculated through the “Inhibition index” (inhibition index = 100 - cell viability). This calculation was based on the response surface model and the highest single agent (HSA) calculation method (Ianevski et al. 2020). HSA synergy scores>0 indicated synergism (represented by red regions), with scores>10 denoting strong synergy. Heatmaps depicting drug combination responses were employed to assess the therapeutic implications of these combinations.

### Migration and invasion assays

To assess the migratory and invasive potential of PC cells, experiments were conducted using various techniques. Initially, PC cells in an optimal growth state were cultured in 6-well plates until reaching full confluency. A straight-line artificial wound was then introduced using the tip of a 200µL pipette, and wound healing progress was monitored under a microscope at 0 h and 48 h at identical locations. The wound healing rate was subsequently quantified using Image Pro Plus 6.0 software (Media Cybernetics, Inc., USA) to represent the migratory capacity of the cells. For another migration experiments, 4 × 10^4^ PC cells suspended in 400 µl of serum-free medium were seeded in the upper chamber of 8µM pore polycarbonate membrane filters (Millipore, USA). To assess invasion capabilities, Matrigel (BD Biosciences, USA) were pre-coated on the upper chamber of the filters 4 hours prior to cell seeding. The lower chamber was filled with complete medium containing 20% fetal bovine serum (FBS) to facilitate cell migration. Following 24–36 h of incubation, the cells were fixed, stained with crystal violet, and viewed under a microscope. Three random fields of view were selected for each group to count the cells, allowing for a comprehensive evaluation of the migratory and invasive behavior of PC cells.

### Flow cytometry detection

After inoculating cells in 6-well plates at a concentration of 5 × 10^4^ per well, appropriate drugs were administered 12 h later. Subsequently, a 48 h incubation period was allowed before the cells underwent digestion with EDTA-free trypsin. Apoptosis levels were evaluated by utilizing the apoptosis kit from BD Biosciences (San Jose, CA, USA). Following the manufacturer’s protocol, the cells were labeled with specific fluorescent dyes for analysis. The quantification of the percentage of apoptotic cells at each stage was conducted using the NovoCyte Flow Cytometer (Agilent, CA, USA).

### Detection of ROS level

After incubating PC cells in 6-well plates at a density of 1 × 10^4^ cells per well for 12 h, the selected drugs were added. Following a 48 h incubation period, cells were labeled with an ROS assay kit (Beyotime, Guangzhou, China) as the manufacturer’s instructions. The cells were then examined using fluorescence microscopy (Carl Zeiss, Oberkochen, Germany) to determine the level of ROS. Subsequently, the cells post-intervention was digested using EDTA-free trypsin, labeled with the same ROS assay reagent, and analyzed for ROS production levels using NovoCyte flow cytometry.

### Detection of intracellular Cu^2+^, glutathione (GSH) and malondialdehyde (MDA) level

PC cells were inoculated in 6-well plates at 5 × 10^4^ per well, followed by the addition of the appropriate drug interventions after 12 h. Subsequently, after 48 h, the levels of intracellular Cu^2+^, GSH, and MDA were evaluated using the Cu^2+^ assay kit (Elabscience, Wuhan, China), the GSH assay kit (Beyotime, Guangzhou, China), and the MDA assay kit (Beyotime, Guangzhou, China), respectively, in accordance with the provided manufacturer’s instructions.

### Western blotting assay

Total cellular proteins were extracted and separated by electrophoresis using SDS-PAGE gels, followed by electroblotting to PVDF membranes (Roche, Penzberg, Germany). The membranes were blocked using QuickBlock™ Blocking Buffer for 20 min, incubated overnight at 4 °C with the primary antibody, washed three times with PBST, and then incubated with a secondary antibody for 1 h at room temperature. Subsequently, the membranes were washed again, and the expression of proteins was detected using a ChemiDoc XRS System (Bio-Rad, CA, USA) with an enhanced chemiluminescence (ECL) kit (NCM Biotech, Suzhou, China).

### Quantitative real-time PCR (qRT–PCR)

Total RNA was extracted from PC cells using the Fastgen2000 RNA isolation system (Fastgen, Shanghai, China) following the manufacturer’s instructions. The extracted RNA was then reverse-transcribed into cDNA using a PrimeScript RT reagent kit (TaKaRa, Dalian, China). Subsequently, quantitative real-time PCR (qRT–PCR) were conducted using a Bio-Rad iQTM5 system (Bio-Rad) to analyze the gene expression levels. The qRT–PCR data was analyzed utilizing the 2^−ΔΔCt^ method. The primer sequences used in this study were as follows: DLAT-F: CAGCTACT-CCTGCTGGACCAAA, DLAT-R: GGTGATTCTACCATCTGGTCCTG, FDX1-F: CTGGCTTGTTCAACCTGTCACC, FDX1-R: GATTTGGCG-CAACCGTGAT, GAPDH-F: GCCAAGAAGGTTCAGCCTGATG, and GAPDH-R: GTCTACATCTGCCTCACGAAGTG.

### Immunofluorescence (IF) assay

The PC cells received suitable interventions in 24-well plates overnight. Next, cells were washed three times with PBS before mitochondria were labeled using the Mito-Tracker kit (Beyotime, Guangzhou, China) as the provided instructions for 30 min. Subsequently, the cells were fixed for 20 min and then permeabilized with 0.5% Triton X-100 (Sigma-Aldrich). Following this, cells were blocked with 1% BSA for additional 1 h and incubated with the relevant primary antibody overnight. Then cells were incubated with CoraLite 488/594-coupled secondary antibodies (Thermo Fisher Scientific, USA) for 1 h at 25 °C. After another round of washing with PBS, the nuclei were stained with 4,6-diamidino-2-phenylindole (DAPI) (Sigma-Aldrich, Germany). Finally, inverted fluorescence microscopy (Carl Zeiss, Oberkochen, Germany) was employed for the acquired of images.

### Autodock model

The AutoDock (Forli et al. 2016) software was employed to assess the spatial geometry and energy compatibility between ligands and receptors. This approach facilitates the prediction of binding conformations and free energies for small molecule ligands targeting larger molecules. The three-dimensional structural models of proteins and their ligands are sourced from the RCSB Protein Data Bank (https://www.rcsb.org/). The protein monomer structures are processed using PyMOL software (San Carlos, California, USA) (Seeliger and Groot 2010). The three-dimensional structural models of small molecules are obtained from PubChem (https://pubchem.ncbi.nlm.nih.gov/) and processed using Obgui software. Finally, PyMOL is utilized to visualize the docking results from AutoDock, highlighting the action patterns and spatial structures of the docking model.

### Tumor xenograft model

Four-week-old female BALB/c nude mice were selected for the tumor xenograft model using the PC cell line MIA PaCa-2. Initially, a density of 4 × 10^7^/mL cells were mixed with Matrigel (BD Biosciences, USA) at a 1:1 ratio. Subsequently, each mouse was injected subcutaneously with 100 uL of the cell suspension. After one week, the mice were randomly allocated into 4 groups: Control (NC), CHL, GEM and CHL combined with GEM. Specific drug interventions were administered at predetermined time intervals, and tumor size as well as body weight was measured twice a week throughout the study. Following three weeks of drug administration, the mice were anesthetized, and blood samples were collected from the eyeballs for the assessment of liver and kidney function in serum. Afterward, the mice were euthanized, and the subcutaneous tumors were excised, weighed, and their volume calculated using the formula: length × width^2^ × 0.52. Subsequently, organs including the heart, liver, spleen, lung and kidneys were harvested from the mice, fixed alongside the tumor tissues, and subjected to Hematoxylin and eosin (H&E) staining for histological analysis. Furthermore, the tumor tissues were stained with TdT-mediated dUTP nick-end labeling (TUNEL) fluorescence staining to assess apoptosis. All animal procedures were performed in strict compliance with the guidelines and protocols approved by the Animal Ethics Committee of the Health Science Center of Xi’an Jiaotong University (No. XJTUAE2023-2174).

### Statistical analysis

All statistical analyses were conducted using SPSS version 21 software (SPSS, Chicago, IL, USA), with experimental data reported based on a minimum of three replications. Student’s two-tailed t-test was used to compare differences between two groups, while one-way ANOVA was employed for comparisons among multiple groups (≥ 3). LSD-t test was used for further two-by-two comparisons between specific groups. Statistical significance was defined as *P* < 0.05.

## Results

### CHL and GEM exert synergistic effects in inhibiting the proliferation of PC cells

**Fig. 1:**
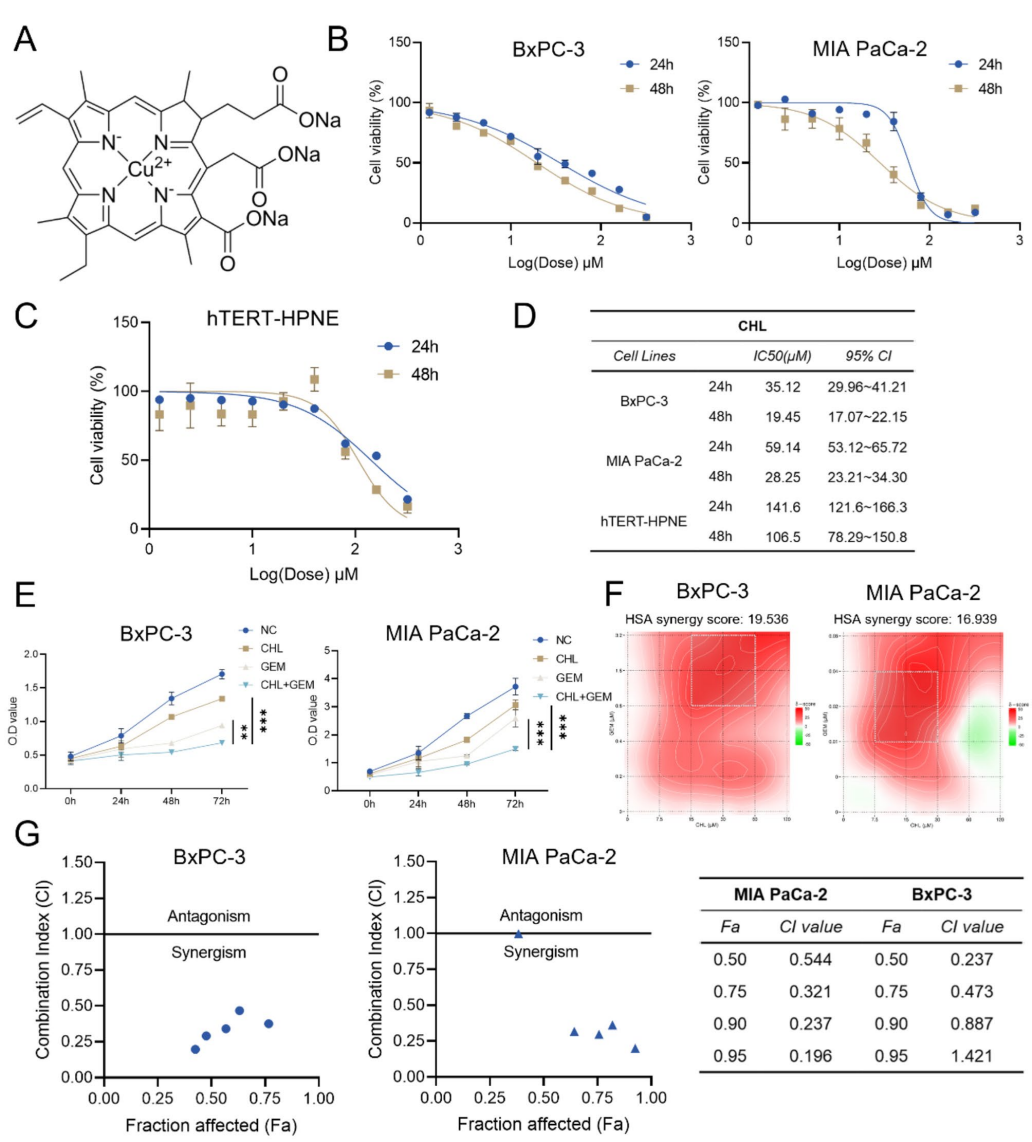
CHL and GEM exert synergistic effects in inhibiting the proliferation of PC cells. **(A)** Chemical structure of CHL. **(B)** Various concentrations of CHL were used to treat MIA PaCa-2, BxPC-3 and **(C)** hTERT-HPNE cells for 24 h and 48 h, and cell viability was determined using a CCK-8 assay. **(D)** The IC50 values of CHL treatment in all cells were analyzed. **(E)** CHL and GEM, alone or in combination, were used to treat MIA PaCa-2 and BxPC-3 cells for different times. Cell proliferation was detected using OD value. **(F)** Heatmaps of drug combination responses. CHL and GEM act synergistically on MIA PaCa-2 and BxPC-3 cells. HSA Synergy scores were calculated using Synergyfinder software, and the white rectangle indicates the concentrations encompassing the region of highest synergy. **(G)** The combination of CHL and GEM showed synergistic effect in MIA PaCa-2 and BxPC-3 cells cell lines. Combination Index was analyzed by CompuSyn Software, and CI values < 1.0 demonstrated that the combinations are synergistic. **p < 0.01, ***p < 0.001

As a semisynthetic derivative of chlorophyll, the anticancer properties of CHL have been reported in several cancers, including PC (Vankova et al. 2018). The structure of CHL is illustrated in Fig. 1A. We first investigated its therapeutic potential on cell proliferation in MIA PaCa-2 and BxPC-3 cells, two commonly used PC cell lines. As observed through the CCK-8 assay, CHL demonstrated a noteworthy inhibitory effect on the proliferation of these PC cells (Fig. 1B). Interestingly, the impact of CHL on the proliferation of pancreatic ductal epithelial cells hTERT-HPNE was less pronounced (Fig. 1C). Notably, the IC50 of CHL against hTERT-HPNE cells was approximately 5-fold higher than that of BxPC-3 cells and 4-fold higher than that of MIA PaCa-2 cells, indicating its potential safety for in vitro applications (Fig. 1D). Consequently, we applied a uniform CHL concentration of 30 µM to intervene with PC cells in subsequent experiments based on the IC50 results.

By comparing the inhibitory abilities of CHL and GEM on the proliferation of PC cells, we observed that CHL had a weaker inhibitory effect than GEM, as indicated by the IC50 values differing significantly (Fig. S1). Therefore, we hypothesized that combining these two drugs might yield a synergistic anti-tumor effect. Subsequently, PC cells were treated with a combination of CHL and GEM at their respective IC50 concentrations. Interestingly, the results of this combination treatment exhibited significantly (*P* < 0.01) enhanced inhibition of cell proliferation compared to treatment with monotherapy, as evidenced in both MIA PaCa-2 and BxPC-3 cells (Fig. 1E). Notably, the antiproliferative effects of CHL and GEM in combination exhibited a concentration-dependent response. Utilizing SynergyFinder software, the drug interaction profiles were analyzed, revealing that the average proportions of the anti-tumor response attributed to the drug combination (HSA synergy score) were 19.536 in BxPC-3 cells and 16.939 in MIA PaCa-2 cells (Fig. 1F). These results strongly suggested a highly synergistic effect of CHL and GEM in suppressing tumor cell proliferation, as evidenced by the synergistic HSA scores >10. Furthermore, employing the Chou-Talalay method via CompuSyn software, the Combination Index (CI) values were determined to be < 1 (indicative of synergism) in both cell lines (Fig. 1G). These findings underscored the synergistic enhancement of cytotoxicity against PC cells by the combination of CHL and GEM at considerably lower IC50 values.

### CHL and GEM synergistically inhibit the oncogenic growth, migration and invasion, and inducing apoptosis of PC cells

**Fig. 2:**
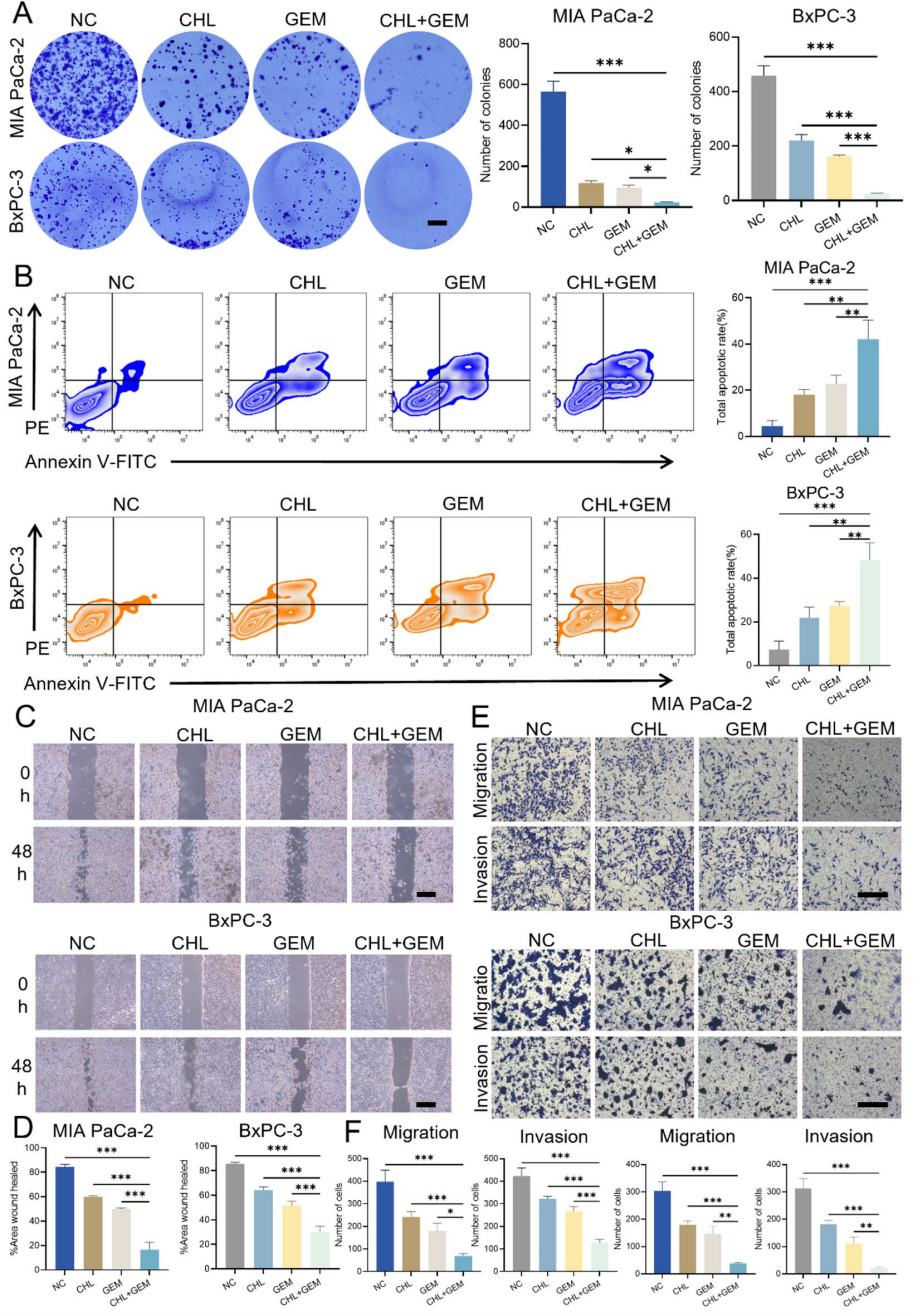
CHL and GEM synergistically inhibit the oncogenic growth, migration and invasion, and inducing apoptosis of PC cells. CHL, GEM or their combination were used to treat MIA PaCa-2 and BxPC-3 cells, and **(A)** cell proliferation were measured by a colony formation (scale bar: 5 mm); **(B)** the cell apoptosis was analyzed by a flow cytometry detection; **(C) and (D)** wound healing assay was carried out to detect cells’ migratory ability (scale bar: 500 μm); **(E) and (F)** transwell migration and invasion assay was conducted to evaluate cells’ migratory and invasive ability (scale bar: 200 μm). Data are presented as mean ± SD of three separate experiments. *p < 0.05, **p < 0.01, ***p < 0.001. *PE* Phycoerythrin

To explore further anti-tumor activity beyond the growth, we conducted a colony formation assay on MIA PaCa-2 and BxPC-3 cells. Initial assessments revealed that the individual application of CHL or GEM moderately suppressed colony formation. However, their combined administration resulted in a significantly more pronounced reduction in the number of colonies formed (*P* < 0.05) (Fig. 2A). Apoptosis induction in cancer cells is crucial for understanding anticancer mechanisms. Therefore, to assess the potential of CHL and GEM in promoting apoptosis in PC, flow cytometry analysis was employed in MIA PaCa-2 and BxPC-3 cells following treatment with the two agents. Our findings demonstrated that the combination of CHL and GEM led to a substantial increase in tumor cell apoptosis compared to when used individually (*P* < 0.01) (Fig. 2B).

Next, we evaluated the effects of the combination of CHL and GEM on the invasion and migration of PC cells. As a highly aggressive tumor, PC frequently undergoes distant metastasis and local infiltration, which can be attributed to the increased invasion and motility of PC cells. Firstly, through the wound healing assay, we observed a significantly larger wound healing area % in PC cells under the treatment of both drugs compared to treatment with CHL or GEM alone (*P* < 0.001) (Fig. 2C-D). Additionally, to comprehensively assess the impact of the combination on the invasive and migratory abilities of PC cells, we conducted a transwell assay. Our findings showed that while CHL or GEM alone only moderately inhibited the invasion and migration of PC cells, the combination of the two drugs significantly enhanced this inhibition (*P* < 0.05) (Fig. 2E-F). Taken together, our results indicate that the synergistic effect of CHL combined with GEM effectively inhibits the malignant properties of PC cells, leading to induced cell apoptosis.

### CHL alters the redox state of PC cells and increases intracellular Cu^2+^ levels

**Fig. 3:**
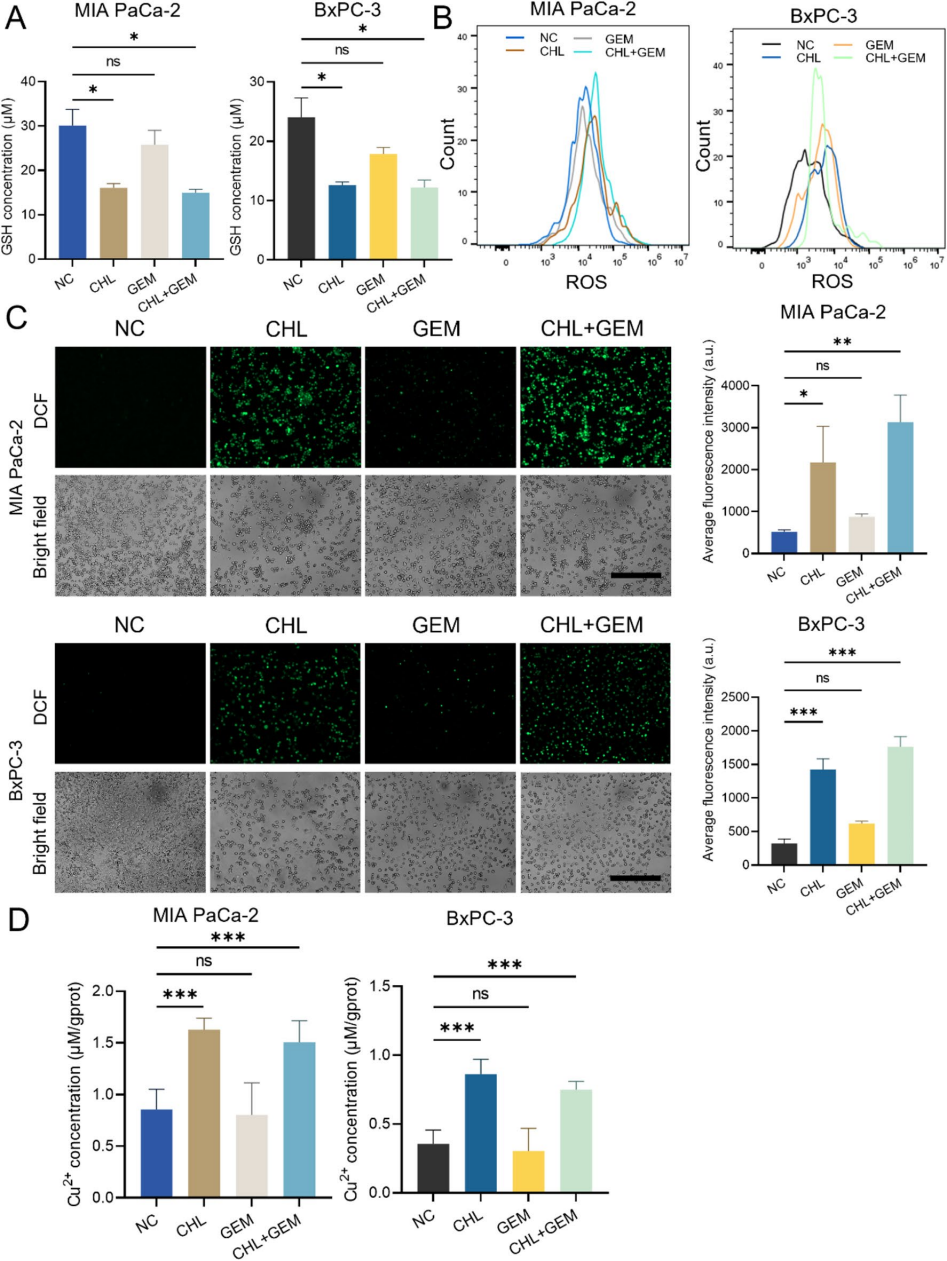
CHL alters the redox state of PC cells and increases intracellular Cu^2+^ levels. CHL, GEM or their combination were used to treat MIA PaCa-2 and BxPC-3 cells, and **(A)** the concentration of GSH were detected using a GSH assay kit; the level of ROS was analyzed using **(B)** flow cytometry and **(C)** fluorescence microscopy (scale bar: 500 μm); **(D)** the concentration of Cu^2+^ was detected using a Cu^2+^ assay kit. Data are presented as mean ± SD of three separate experiments. *p < 0.05, **p < 0.01, ***p < 0.001, *ns*: Not significant

In order to investigate the molecular mechanisms underlying the synergistic effects of CHL in combination with GEM for the treatment of PC, our initial focus was on understanding the unique properties of CHL. As a copper sodium salt of a chlorophyll derivative, CHL has been previously associated with regulating the redox state in various tumor types (Ozcan et al. 2021; Ozcan et al. 2019). This biological compound is known to influence the dynamics of GSH and ROS levels (Vankova et al. 2018; Sun et al. 2021), potentially playing a crucial role in its therapeutic effects. Subsequently, we conducted an analysis to determine the impact of CHL and GEM treatments on the levels of GSH and ROS within PC cells MIA PaCa-2 and BxPC-3. The data indicated a more significant reduction in GSH levels with CHL treatment compared to GEM (*P <* 0.05), similar results were observed in the CHL combined GEM group (Fig. 3A). For the analysis of ROS levels, we utilized both flow cytometry and fluorescence microscopy. The findings on both cell lines consistently revealed an enhancement in intracellular ROS production by CHL treatment, supported by an increased percentage of ROS-positive cells (Fig. 3B) and stronger fluorescence intensity (*P <* 0.05) (Fig. 3C).

CHL, as a metal-based derivative of chlorophyll, contains Cu^2+^ in its structure, making copper an essential trace element for life. However, excessive copper accumulation can overwhelm a cell, leading to death (Kahlson and Dixon 2022). Nevertheless, an optimum level of intracellular copper accumulation may offer a window for selectively targeting cancer cells (Ge et al. 2022). Tsvetkov et al. (Tsvetkov et al. 2022) revealed that copper toxicity disrupts the tricarboxylic acid cycle, ultimately inducing cell death, thus suggesting a potential use of copper toxicity in cancer treatment strategies. To investigate the therapeutic effects of CHL on PC in relation to copper toxicity, we treated PC cells with CHL, GEM, or their combination and assessed intracellular Cu^2+^ levels. Our results demonstrated a significant elevation in intracellular Cu^2+^ concentration in the CHL and CHL combined GEM treated group (*P* < 0.01) (Fig. 3D), but not with GEM treatment. In summary, CHL modifies the redox environment of PC cells and triggers copper toxicity by increasing intracellular Cu^2+^ levels.

### CHL binds to FDX1, promotes DLAT oligomerization and oxidative stress in PC cells

**Fig. 4:**
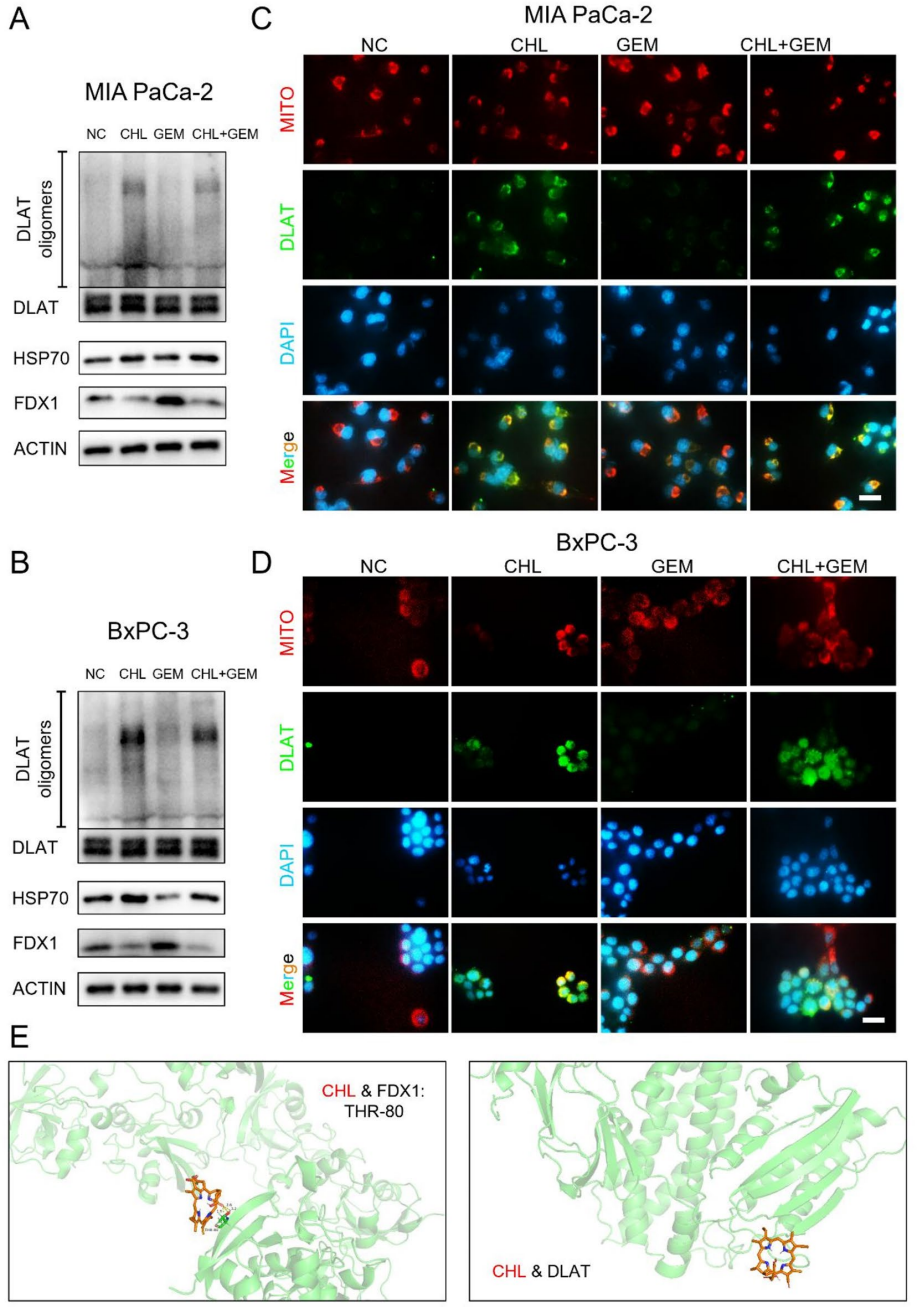
CHL binds to FDX1, promotes DLAT oligomerization and oxidative stress in PC cells. **(A)** MIA PaCa-2 cells and **(B)** BxPC-3 cells were treated with CHL, GEM or their combination, and levels of FDX1, HSP70, DLAT and DLAT oligomers were detected by Western blotting analysis. After treated with CHL, GEM or their combination, the morphology, expression and localization relative to mitochondria of DLAT in **(C)** MIA PaCa-2 and **(D)** BxPC-3 cells were demonstrated by immunofluorescence assay. The Mito-Tracker Deep Red FM (red), DLAT antibody (green), and 4, 6-diamidino-2-phenylindole (DAPI) (blue) was used for staining mitochondria, DLAT and the nucleus, respectively (scale bar: 50 μm). **(E)** AutoDock model of CHL and DLAT interaction with FDX1. The yellow dotted line and labels: hydrogen bonds and distance of the hydrogen bond

Firstly, Tsvetkov et al. (Tsvetkov et al. 2022) demonstrated that excess copper accumulation selectively interferes with a group of mitochondrial lipoylated metabolizing enzymes, notably DLAT, thereby affecting cellular activity. They also found that the mitochondrial enzyme FDX1 is crucial for lipoic acid synthesis and disruption of this enzyme leads to inhibition of copper-dependent toxicity. FDX1 serves as an essential upstream molecule in copper-mediated cytotoxicity, with DLAT and the oxidative stress protein Heat Shock Protein 70 (HSP70) acting as key effector molecules. Therefore, we performed a western blotting analysis to investigate the alterations in protein levels linked to copper-induced cytotoxicity in MIA PaCa-2 and BxPC-3 cells at 48 h. Our findings demonstrated that CHL significantly decreased the levels of FDX1 protein, while concurrently increasing the levels of HSP70 protein in both cell lines (Fig. 4A-B). Although the protein levels of DLAT did not escalate under CHL intervention, there was a notable augmentation in protein oligomerization. Notably, we found GEM treatment yielding no significant effects in comparison. Remarkably, our investigation revealed that neither CHL nor GEM had any impact on the mRNA expression levels of FDX1 and DLAT (Fig. S2), indicating that the regulatory mechanisms did not transpire at the transcriptional level.

To validate this observation, we conducted immunofluorescence assays to visualize the distribution and expression of DLAT in two different PC cells. Intriguingly, we observed that under CHL intervention, the green fluorescence denoting DLAT protein showed aggregation of punctate structures, which was further supported by the enhanced fluorescence intensity (Fig. 4C-D). Similarly, no such changes were found in the GEM intervention group. Based on these insights, we conducted a study to predict the interactions between the small molecular ligand CHL and the macromolecular target FDX1 and DLAT using AutoDock tools. The computational analysis demonstrated that CHL and FDX1 can form hydrogen bonds with THR-80 in the kinase domain of c-Met, exhibiting a calculated binding energy of -4.35 kcal/mol. However, no binding site was identified between CHL and DLAT (Fig. 4E). This suggests that CHL may act by binding FDX1 but not DLAT, which subsequently decreased the FDX1 protein levels. Collectively, these outcomes suggest that by binding to FDX1 in PC cells, CHL can induce copper-mediated cytotoxicity.

### CHL induces Cuproptosis in PC cells

**Fig. 5:**
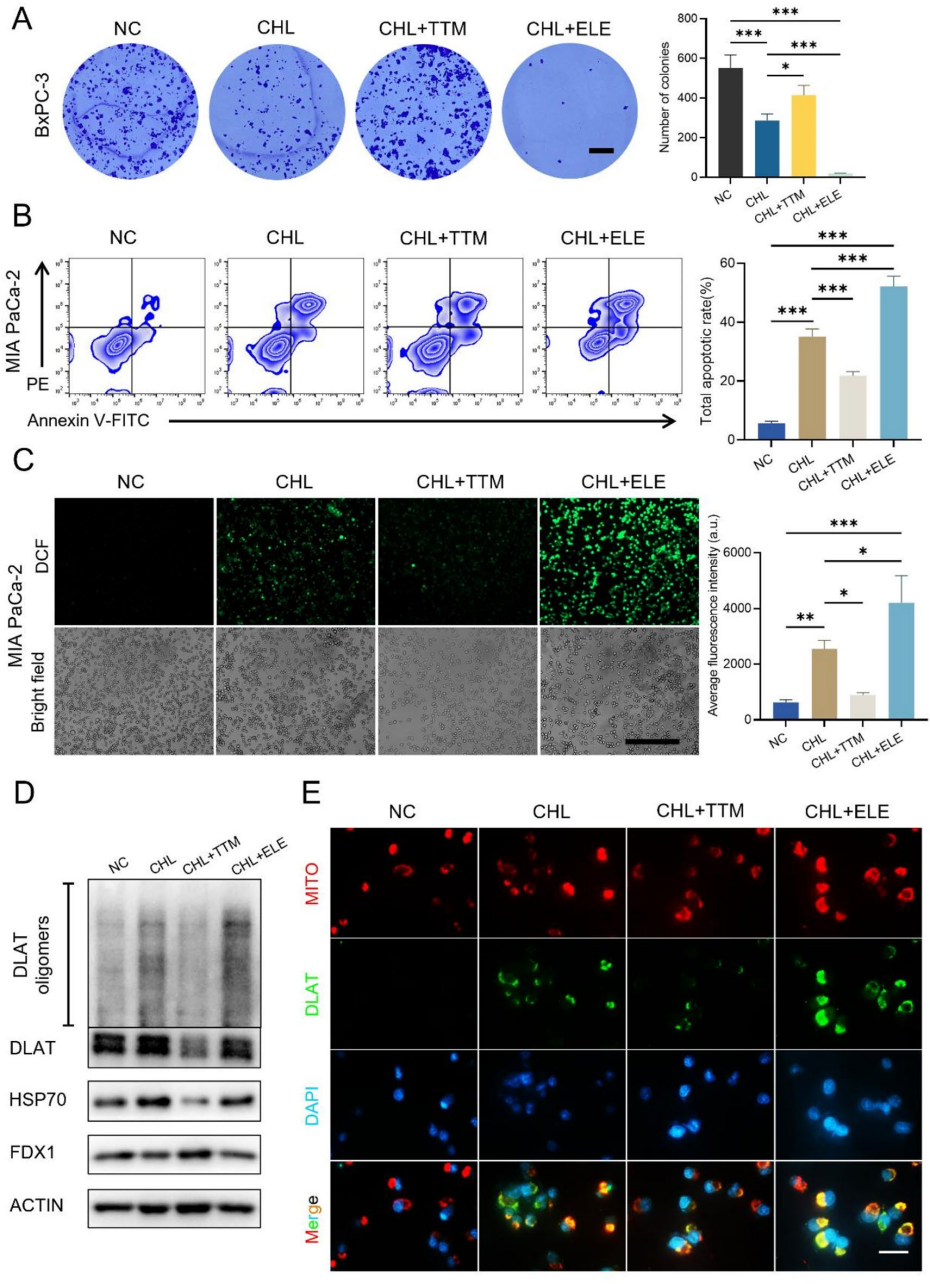
CHL induces cuproptosis in PC cells. **(A)** BxPC-3 cells were treated with CHL, CHL combined with ELE (a cuproptosis activator) and CHL combined with TTM (a cuproptosis inhibitor), cell proliferation was measured by a colony formation (scale bar: 5 mm). MIA PaCa-2 cells were treated with CHL, CHL combined with ELE and CHL combined with TTM, and **(B)** cell apoptosis was analyzed by a flow cytometry detection; **(C)** level of ROS was analyzed using flow cytometry (scale bar: 500 μm); **(D)** levels of FDX1, HSP70, DLAT and DLAT oligomers were detected by Western blotting analysis; **(E)** the morphology, expression and localization relative to mitochondria of DLAT were demonstrated by immunofluorescence assay (scale bar: 100 μm). Data are presented as mean ± SD of three separate experiments. *p < 0.05, **p < 0.01, ***p < 0.001. *ELE* Elesclomol, *TTM* Ammonium tetrathiomolybdate (VI)

Next, we examined whether the amplified sensitizing effect of CHL on GEM was related to the promotion of cuproptosis. As a recently characterized form of programmed cell death, cuproptosis is characterized by the accumulation of lipoylated proteins, enhanced oxidative stress, and a central role of copper ions (Tsvetkov et al. 2022). Studies have shown that copper ionophores, like elesclomol (ELE), can sequester copper and transport it into cells to trigger cuproptosis, whereas copper chelators such as Ammonium tetrathiomolybdate (TTM) and GSH can protect against copper-induced cuproptosis. To investigate this, PC cells were treated with CHL, CHL combined with ELE, and CHL combined with TTM. The results from colony formation (Fig. 5A) and flow cytometry (Fig. 5B) indicated that CHL not only inhibits oncogenic growth and induces apoptosis in PC cells but also that this effect is augmented by ELE and partially mitigated by TTM.

We then investigated the levels of GSH and ROS in PC cells following combined ELE and TTM interventions, revealing that ELE increased ROS production within the cells (Fig. 5C) while TTM partially counteracted this effect. Notably, the interventions had minimal impact on GSH depletion (Fig. S3). Additionally, our western blotting analysis indicated that ELE heightened the protein levels of HSP70 and promoted the oligomerization of DLAT proteins beyond what was observed in the single CHL treatment group. In contrast, TTM mitigated these alterations to some extent (Fig. 5D). Subsequent immunofluorescence assays validated these effects on DLAT proteins (Fig. 5E). These findings support the conclusion that the sensitization of PC to GEM chemotherapy by CHL involved cuproptosis pathways, as confirmed by the use of cuproptosis promoters and inhibitors.

### CHL and GEM synergistically inhibit PC tumor growth in vivo with potential biosafety

**Fig. 6:**
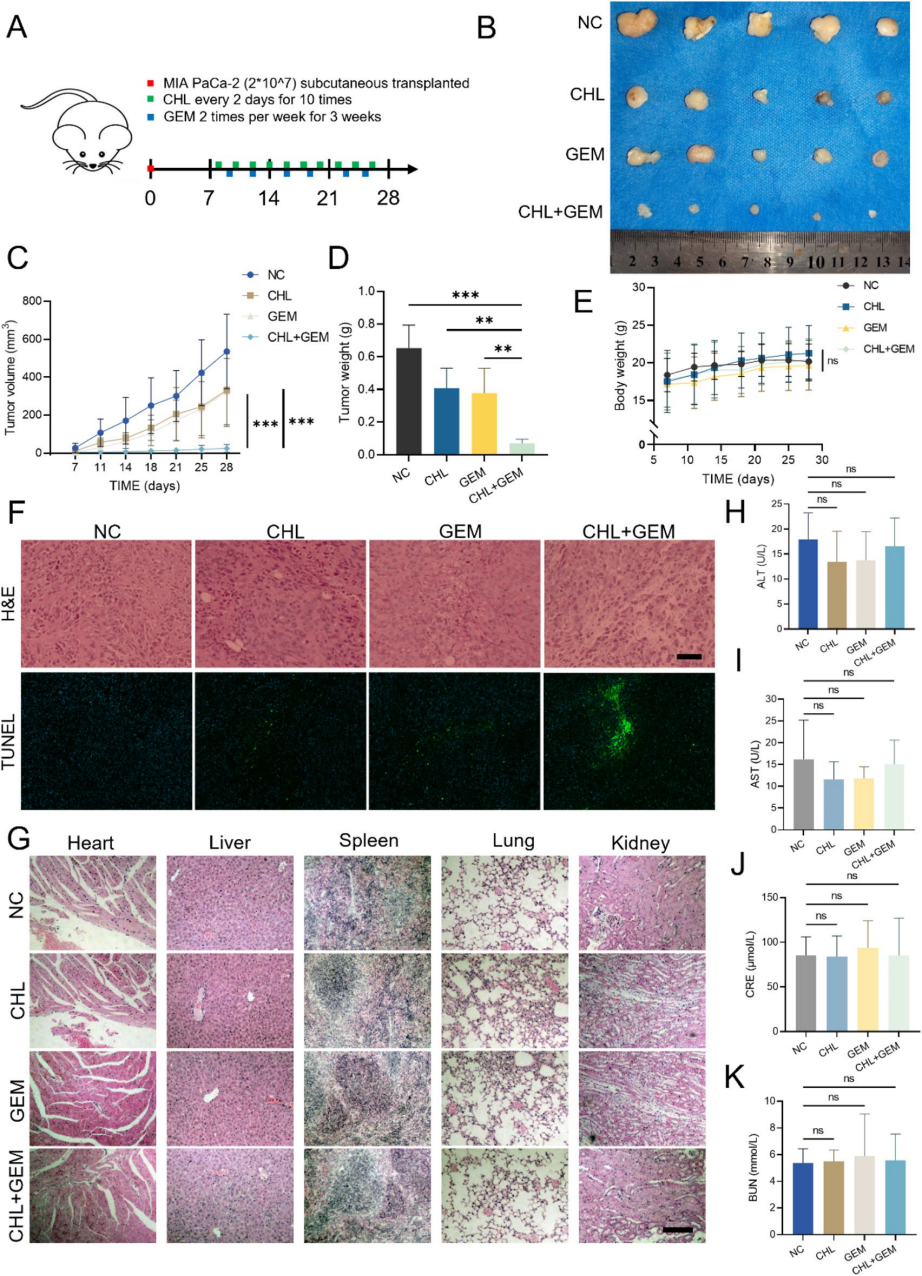
CHL and GEM synergistically inhibit PC tumor growth in vivo with potential biosafety. **(A)** The subcutaneous xenograft model mice with MIA PaCa-2 cells were treated with CHL, GEM, alone or in combination, or with the control (saline control). The tumor volumes and body weight were measured twice a week until the experiment was terminated. **(B)** Representative images of the subcutaneous xenograft tumor. **(C)** The changes in tumor volume of the mice receiving different treatments. The average weights of the tumors **(D)** and changes in body weight of mice **(E)** after different treatments. **(F)** H*&*E and TUNEL staining of the indicated xenograft tumors (scale bar: 500 μm), **(G)** H*&*E staining of heart, liver, spleen, lung and kidney from mice receiving different treatments (scale bar: 200 μm). Serum levels of **(H)** ALT, **(I)** AST, **(J)** CRE, and **(K)** BUN were determined in mice from different treatment groups. Data are presented as mean ± SD of three separate experiments. **p < 0.01, ***p < 0.001, *ns*: Not significant. *H&E* Hematoxylin and eosin, *TUNEL* TdT-mediated dUTP Nick-End Labeling, *ALT* Alanine transaminase, *AST* Aspartate aminotransferase, *CRE* Creatinine, *BUN* Blood urea nitrogen

In order to assess the anti-tumor efficacy and safety profile of combining CHL and GEM in vivo, a MIA PaCa-2 xenograft model was established in nude mice. The mice with tumors were randomly allocated into four groups, each consisting of five mice, and were administered intraperitoneal injections as follows: saline (control group, administered once every two days), CHL group (once every two days), GEM group (twice per week), and CHL combined with GEM group (same frequency as the single-agent groups) for a duration of three weeks (Fig. 6A). The results indicated that both CHL and GEM monotherapies resulted in a certain level of tumor growth inhibition. However, the combination therapy of CHL and GEM demonstrated a more pronounced anticancer effect when compared to either drug alone (Fig. 6B-D, S4-S5). Notably, the body weight of all treated mice did not show a significant change in comparison to the control group (Fig. 6E and S6), suggesting that the combination treatment of CHL and GEM was well tolerated by the experimental animals.

To further confirm the efficacy of CHL combined with GEM in inhibiting tumor growth, we conducted H&E and TUNEL staining on mouse tumor tissues. The combined treatment group showed the highest level of membrane fragmentation, nuclear condensation, and strongest fluorescent signals indicative of apoptotic cells (Fig. 6F). To evaluate potential systemic toxicity, we conducted hematoxylin and eosin (H&E) staining on major organs (heart, liver, spleen, lungs, and kidneys) from all treatment groups. Histopathological analysis revealed no detectable structural abnormalities or pathological changes in any of the examined organs (Fig. 6G). Subsequently, we analyzed the treated serum from mice to evaluate liver and kidney function. Our results indicated that the levels of ALT, AST, CRE, and BUN in the treatment group did not show significant alterations compared to the control group (Fig. 6H-K), suggesting no notable liver or renal dysfunction in the drug-administered mice. Collectively, these findings validate the anti-tumor efficacy and safety profile of CHL combined with GEM in an in vivo setting.

## Discussion

As one of the most malignant cancers, the incidence of PC is increasing annually, making it a significant public health concern. Despite the continuous evolution of diagnostic methods and therapeutic strategies (Wei and Ren 2024), the prognosis for PC patients remains poor. While surgery and chemotherapy are still the primary treatment options for PC, surgical intervention is feasible in only a limited percentage of patients (15% ~ 20%) at the time of diagnosis (Kleeff et al. 2016). This highlights the vital role of chemotherapy in the postoperative adjuvant and palliative treatment of the disease. Among the essential chemotherapeutic agents for PC, GEM stands as the cornerstone of treatment. However, the efficacy of GEM has been compromised by the development of chemoresistance. Additionally, the severe toxicity associated with GEM combination therapy poses challenges for PC patients with progressive disease. Consequently, there is an urgent need to explore effective GEM chemo-sensitization strategies and develop new, low-toxicity treatments for PC.

Combination therapy is a valuable approach aiming to boost anti-tumor efficacy while mitigating the possible adverse effects of high doses of drugs when administered individually. Furthermore, this approach can help prevent the emergence of drug resistance resulting from varied mechanisms of drug action. Combination therapy strategies with GEM encompass concurrent induction of apoptosis (Luo et al. 2021), targeted inhibition of estrogen-related receptor alpha (ERRα) signaling (Liu et al. 2022), and modulation of epigenetic regulation mechanisms (Maietta et al. 2022). In our present study, CHL, a traditional redox modulator and potential anticancer agent, was utilized to enhance the anti-tumor activity of GEM. Reports on CHL mainly document its anti-tumor effects in breast cancer (Ozcan et al. 2019, 2021), lung cancer (Das et al. 2016), and hematologic tumors (Suryavanshi et al. 2015), with limited evidence of its application in PC (Vankova et al. 2018). Notably, our experiments initially illustrated that CHL inhibits the proliferation of PC cells, while exerting minimal effects on normal pancreatic ductal epithelial cells. Importantly, the synergy between CHL and GEM was demonstrated, demonstrating a notable reduction in the required dosage for individual agents in PC treatment. This promising outcome unveils a novel potential strategy for the adjunct treatment of these patients.

Our results also evidenced that the combination regimen of CHL combined with GEM exerts synergistic effects in inducing apoptosis and inhibiting invasion and migration of PC cells. This finding is significant for the treatment of PC because one of the major hurdles in treating PC lies in overcoming its inherent resistance to chemotherapy (Zeng et al. 2019). It shows that CHL can make PC cells more sensitive to GEM, which in turn exerts a stronger killing effect.

Research into the potential mechanisms by which CHL exerts therapeutic effects in PC has been a focus of our investigation. Previous studies have indicated that CHL can prevent cancer development by modulating oxidative stress and regulating xenobiotic metabolic systems (Gradecka-Meesters et al. 2011). A study by Kateřina et al. (Vankova et al. 2018) highlighted CHL’s ability to inhibit PC cells’ redox state by targeting heme oxygenase (HMOX) activity, both in vitro and in vivo. GEM is been known to induce the accumulation of ROS that can cause DNA damage (Binenbaum et al. 2015). To counteract ROS-induced damage, drug-resistant PC cells can regulate GSH production through transcription factors like NF-E2-related factor-2 (Nrf2) to reduce cytotoxic effects. In addition, Ju (Ju et al. 2015) et al. have shown that silencing Nrf2 leads to increased GSH levels and promotes GEM resistance, while inhibiting GSH enhances GEM sensitivity. Consistent with these findings, our research indicates that CHL decreases GSH levels and increases ROS levels in PC cells. This alteration may underlie CHL’s ability to enhance GEM sensitivity and potentiate its therapeutic effects in drug-resistant PC cells. The decrease in GSH levels and the increase in ROS levels induced by CHL could be a significant mechanism of the two drugs’ synergistical anti-tumor effects in PC.

Our results demonstrated that upon entry into PC cells, CHL releases Cu^2+^, leading to an elevated intracellular level of copper. Copper, a metal with dual redox states, plays a crucial role in maintaining enzyme activity and the function of transcription factors in the body (Chen et al. 2022a, b). It exists in two oxidation states within biological systems: Cu^+^ and Cu^2+^. Acting as a catalyst in numerous redox reactions involving cellular, biochemical, and regulatory functions, excess copper concentrations beyond physiological requirements can be cytotoxic (Chen et al. 2022a, b). Studies have shown that it can induce cell death and accelerate cancer cell death by triggering non-apoptotic programmed death (Wang et al. 2024). As a result, cuproptosis, a metal ion-dependent regulated cell death mechanism that relies on intracellular copper, has emerged as a significant consideration in this context.

Recent research by Tsvetkov (Tsvetkov et al. 2022) and colleagues introduced the concept of cuproptosis, showcasing its distinct nature from apoptosis, ferroptosis, and necroptosis. Cuproptosis is characterized by elevated levels of FDX1 and mitochondrial respiration rate, and its progression is contingent upon the availability of copper. Specifically, Tsvetkov (Tsvetkov et al. 2019) et al. observed that copper-induced toxicity disrupts specific mitochondrial metabolic enzymes, as excess intracellular Cu^2+^ being transported to the mitochondria via ionophores. This excess copper is then reduced by FDX1 from Cu^2+^ to Cu^+^. The augmented Cu^+^ content directly interacts with lipoylated DLAT, leading to the aggregation of lipoylated proteins and the destabilization of Fe-S cluster proteins. Subsequently, this cascade of events induces proteotoxic stress, ultimately culminating in cuproptosis (Tsvetkov et al. 2022). Notably, attempts to hinder cuproptosis through genetic or pharmacologic inhibition of apoptosis, ferroptosis, and necrotic apoptosis were ineffective, while the hydrophilic antioxidant GSH suppressed copper-induced toxicity by sequestering intracellular Cu^+^. Our own investigations revealed that CHL binds to FDX1, resulting in increased levels of the oxidative stress protein HSP70 and hastening the aggregation of DLAT protein. Furthermore, we found that the alterations induced by CHL in PC cells can be partially mitigated by the copper death inhibitor TTM and exacerbated by copper ionophores like ELE. Thus, we postulate that the anticancer mechanism of CHL against PC cells may be mediated through cuproptosis.

Given that combination regimens increasingly enhance toxicity levels, the safety of drug combinations also warrants close examination. Over the past decade, two new combination regimens have emerged as first-line treatment options for patients with advanced PDAC. These regimens include the combination of 5-fluorouracil (5-FU), folinic acid, irinotecan, and oxaliplatin (referred to as FOLFIRINOX), as well as the combination of GEM with albumin nanoparticle paclitaxel (nab-paclitaxel) (Hoff et al. 2013; Conroy et al. 2011). Despite their efficacy, both combination regimens present significant challenges to patients’ physical well-being due to their non-negligible side effects during clinical application (Costanzo et al. 2023). Our research candidate CHL, a semi-synthetic derivative of the natural compound chlorophyll, has demonstrated promising synergistic efficacy with GEM against PC in both in vitro and in vivo studies, as well as exhibiting a favorable biosafety profile. The in vivo results indicate that the combined regimen treatment did not exacerbate organ damage in mice and had minimal impact on liver and kidney function. These findings provide essential evidence of drug safety, serving as a potential theoretical groundwork for future clinical investigations.

In addressing the limitations of this study, several key details warrant further attention. Firstly, the signaling pathway through which CHL reduces GSH remains incompletely elucidated. Additionally, the restrictive experimental conditions have hindered a more comprehensive investigation into the intricate mechanisms involving CHL and FDX1. Additionally, the precise role of FDX1 in regulating the reduction of Cu^2+^ to Cu^+^ and the oligomerization processes of DLAT has not been adequately investigated. To address these research gaps, our plan involves systematically conducting a series of detailed and extensive experiments over the next few years to systematically alleviate the aforementioned deficiencies in our experimental approach.

**Fig. 7:**
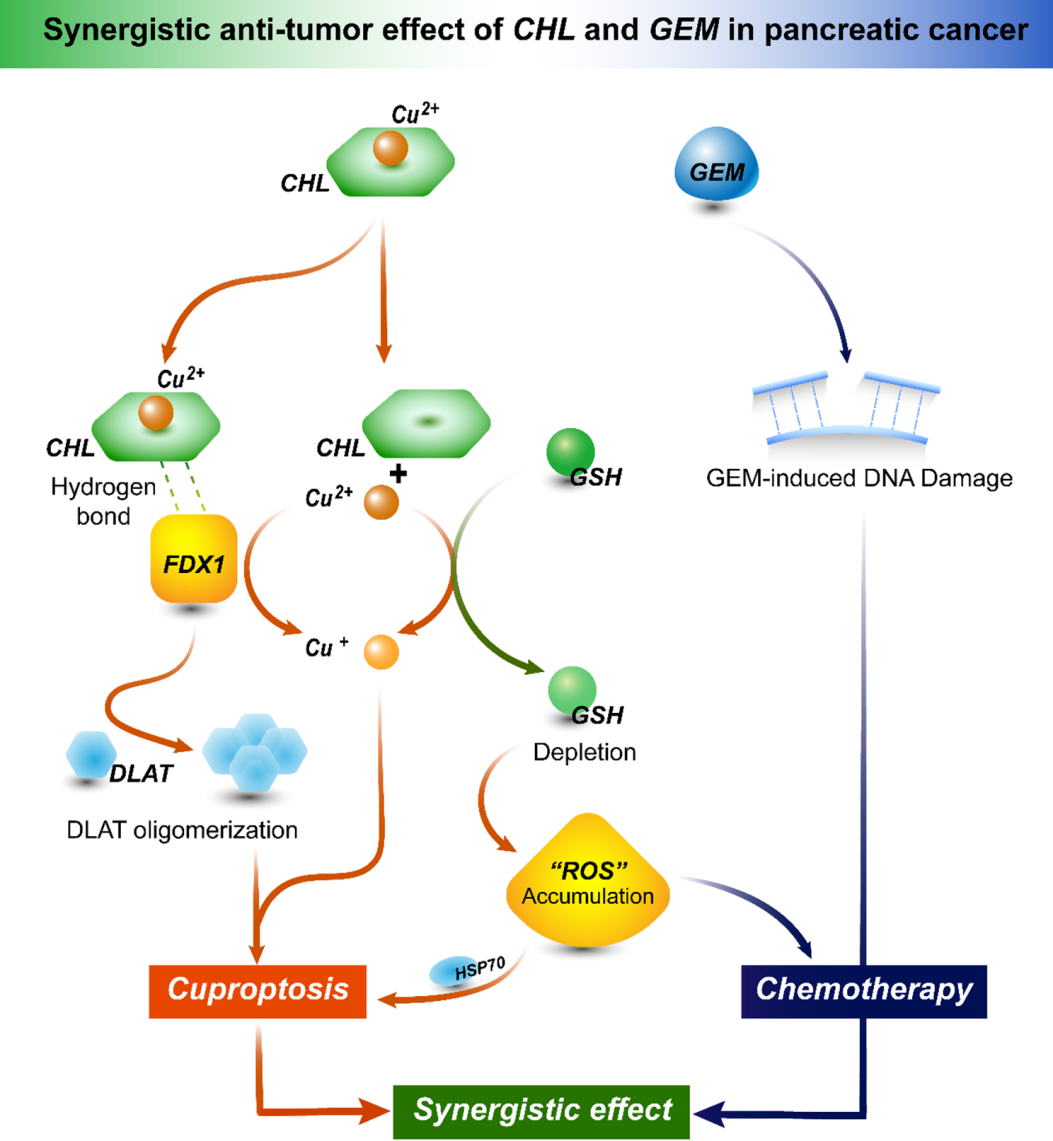
Schematic diagram of the mechanism by which CHL induces cuproptosis and synergizes with GEM in PC. Briefly, CHL induces cuproptosis in PC cells through multiple interconnected pathways: Upon entry into the cell, CHL releases free Cu^2+^ ions, simultaneously cause a decrease in intracellular glutathione (GSH) levels and can bind to FDX1 molecules. The activation of FDX1 reduces Cu^2+^ to Cu^+^ (which increasing cellular copper toxicity) and promotes the oligomerization of DLAT proteins. These processes, combined with the accumulation of reactive oxygen species (ROS) due to GSH depletion, co-induces cuproptosis in PC cells. Additionally, GSH, as a common intracellular reducing agent, can further reduce its own levels by reducing Cu^2+^ to Cu^+^. Interestingly, the reduction in GSH levels amplifies the oxidative stress toxicity of ROS on PC cells, thereby enhancing the chemotherapeutic efficacy of GEM. This phenomenon could underlie the synergistic anti-tumor effects of CHL and GEM in PC

In conclusion, our results suggest that the combined treatment of CHL and GEM offers a synergistic inhibitory effect on the proliferation, invasion, and migration of PC cells. The combined therapy also shows promise in inhibiting tumor growth in PC in vivo, while maintaining a favorable biosafety profile. Furthermore, CHL demonstrates the ability to induce cuproptosis through multiple pathways, and when combined with GEM chemotherapy, it enhances the therapeutic efficacy in treating PC (Fig. 7). These findings highlight the potential of CHL as a novel treatment strategy for PC, emphasizing the need for standard clinical trials to validate its efficacy in the future.

## Electronic supplementary material

Below is the link to the electronic supplementary material.

Supplementary Material 1

## Data Availability

No datasets were generated or analysed during the current study.

## Abbreviations

PC: Pancreatic cancer
GEM: Gemcitabine
CHL: Chlorophyllin
GSH: Glutathione
ROS: Reactive oxygen species
FDX1: Ferredoxin 1
DLAT: Dihydrolipoamide S-Acetyltransferase
TTM: Ammonium tetrathiomolybdate(VI)
ELE: Elesclomol
CI: Combination index
MDA: Malondialdehyde
