# Supplementary material for: Chlorophyllin exerts synergistic anti-tumor effect with gemcitabine in pancreatic cancer by inducing cuproptosis
Source: Mol Med. 2025 Apr 4;31:126. doi: 10.1186/s10020-025-01180-y (PMC11969790; doi:10.1186/s10020-025-01180-y)
Supplement: Supplementary file 1 — Supplementary Material 1 [file 10020_2025_1180_MOESM1_ESM.docx]

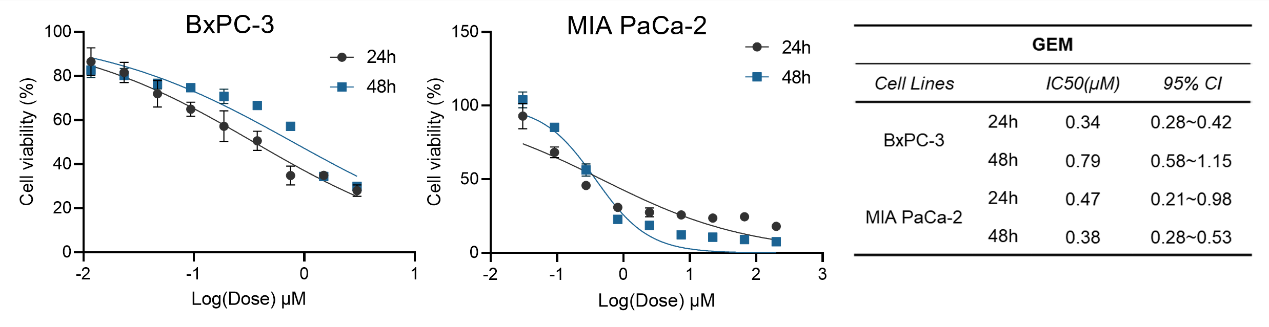


**Fig. S1 The cell viability of PC cells treated with GEM.** BxPC-3 and MIA PaCa-2 cells were treated with various concentrations of GEM for 24h and 48h, and cell viability was determined using a CCK-8 assay. The IC50 values were also analyzed.


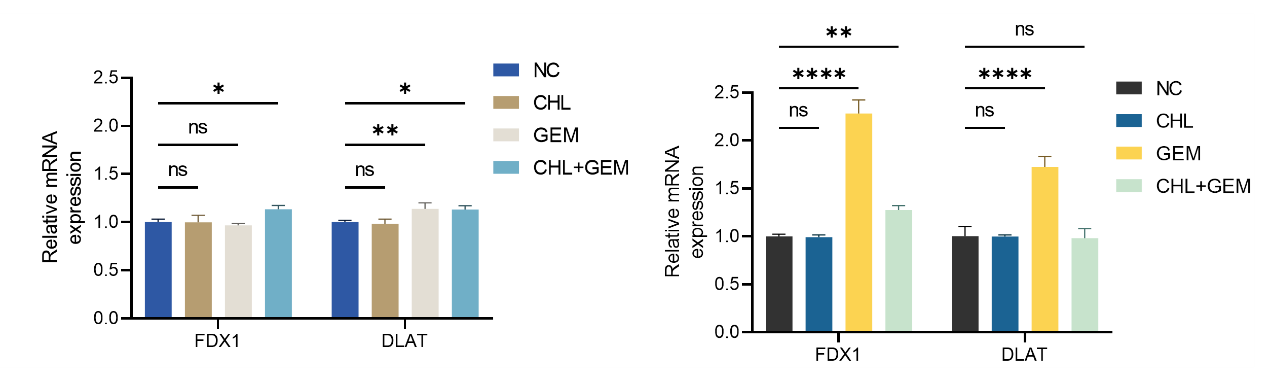


**Fig. S2 Effect of CHL and GEM on the mRNA expression levels of FDX1 and DLAT in PC cells.** CHL, GEM or their combination were used to treat MIA PaCa-2 and BxPC-3 cells**,** and the mRNA expression levels of FDX1 and DLAT were analyzed by a qRT–PCR assay. *ns*: Not significant.


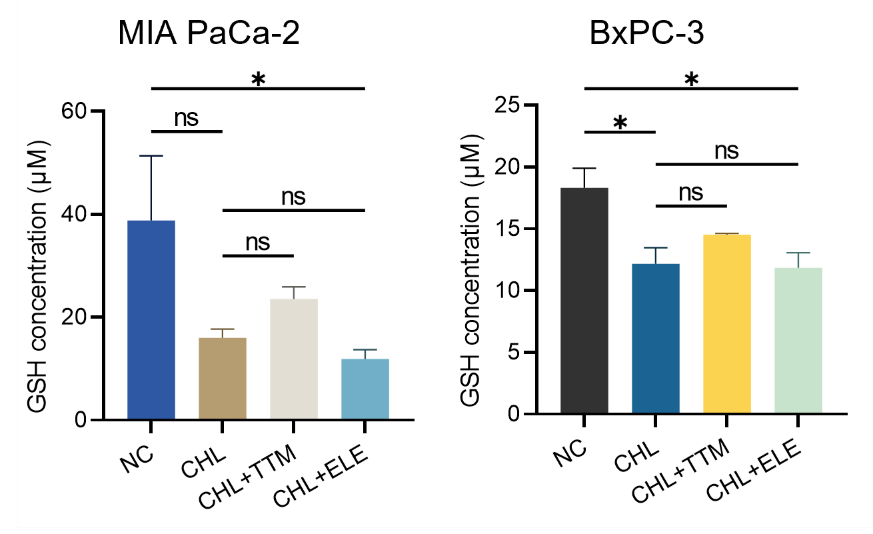


**Fig. S3 The GSH levels in PC cells after treated by CHL combined with ELE/TTM.** CHL, CHL combined with ELE, CHL combined with TTM were used to treat MIA PaCa-2 and BxPC-3 cells**,** and the GSH levels were analyzed by a GSH assay kit. **p* < 0.05, *ns*: Not significant. *ELE* Elesclomol, *TTM* Ammonium tetrathiomolybdate.


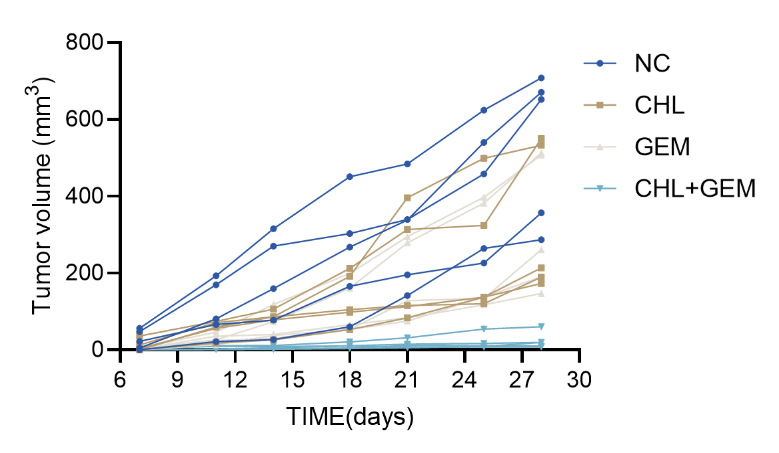


**Fig. S4 The growth curves of tumors in different groups of mice receiving the respective treatments.** The subcutaneous xenograft model mice with MIA PaCa-2 cells were treated with CHL, GEM, alone or in combination, or with the control (saline control). The tumor volumes were measured twice a week until the experiment was terminated.


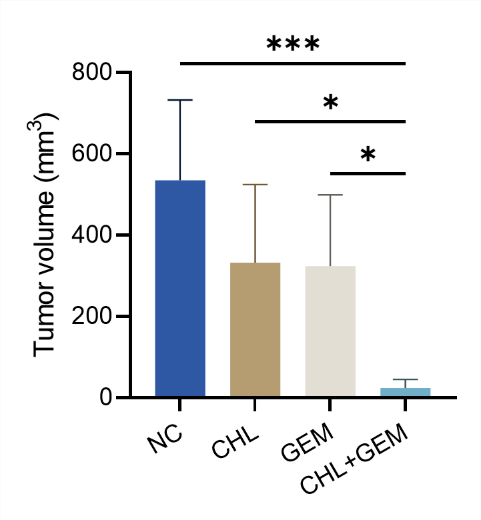


**Fig. S5 Tumor volumes of PC xenografts model mice after treated by CHL combined with GEM.** The subcutaneous xenograft model mice with MIA PaCa-2 cells were treated with CHL, GEM, alone or in combination, or with the control (saline control). The tumor volumes were measured twice a week until the experiment was terminated. **p* < 0.05, ****p* < 0.001.


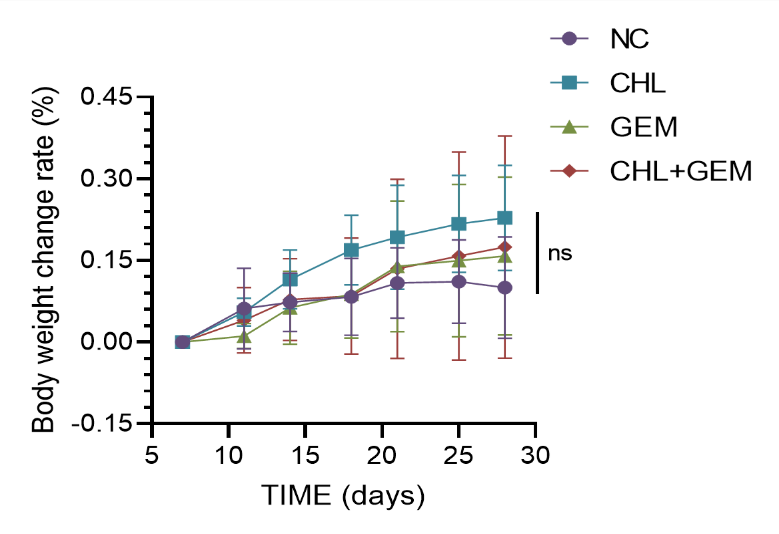


**Fig. S6 Changes in the rate of body weight change in PC xenografts model mice after treated by CHL combined with GEM.** The subcutaneous xenograft model mice with MIA PaCa-2 cells were treated with CHL, GEM, alone or in combination, or with the control (saline control). The body weight was measured twice a week until the experiment was terminated. *ns*: Not significant
